# Heat-related challenges and interventions in hospitals: A future-oriented, qualitative approach to improve nurses' working conditions

**DOI:** 10.1016/j.joclim.2026.100659

**Published:** 2026-04-10

**Authors:** Maria Zink, Andrea Nakoinz, Ulrike Krol, Niels Jansen, Franziska Jung, Steffi G. Riedel-Heller, Katharina M.A. Gabriel

**Affiliations:** a Federal Institute for Occupational Safety and Health (BAuA), Dresden, Germany; b BG Klinikum Unfallkrankenhaus, Berlin, Germany; c KLUG - Deutsche Allianz Klimawandel und Gesundheit e.V., Berlin, Germany; d Ellery Studio, Berlin, Germany; e University of Leipzig, Institute for Social Medicine, Occupational Health and Public Health, Leipzig, Germany; f Federal Institute for Occupational Safety and Health (BAuA), Berlin, Germany

**Keywords:** heatwaves, hospitals, nursing, adaptation, resilience, governance, occupational health, climate, heat stress, interventions, challenges, climate crisis

## Abstract

**Objective:**

This study explores organizational interventions aimed at mitigating heat-related challenges and stress for nurses in acute care settings, with a focus on adapting to the intensifying climate crisis.

**Method:**

Six trans- and interdisciplinary participants participated in the workshop. The one-day workshop was based on the methodology of ‘Zukunftswerkstatt’ (Futures Workshop) and ‘design thinking’ principles. Participants analyzed challenges related to heat waves and developed actionable, future-oriented interventions based on scenarios and tools. Data collection involved photographs of handwritten workshop activities and researchers’ notes. Data analysis followed three main steps to abstract and synthesize results: discussing results using the collected data, participants’ feedback on the workshop documentation, and final synthesis.

**Results:**

The study identified several key interventions to manage heat-related stress, including the development of a comprehensive Heat Health Action Plan (HHAP), modular training programs, and a mobile staff app for real-time communication. Interventions were categorized into four phases: prevention, preparation, response, and recovery, with organizational strategies outweighing individual-level interventions. Participants highlighted the need for leadership commitment, adequate resource allocation, cross-sector collaboration, and clear communication. Successful implementation of HHAP was viewed as dependent on engagement from middle management and its integration into hospital governance and strategic planning.

**Conclusion:**

This study highlights the complexity of heat adaptation in hospitals. Findings underscore the importance of successful heat adaptation for hospital employees. Strengthening institutional commitment and integrating staff-driven approaches are essential for developing robust, future-ready heat preparedness in hospitals.

## 1 Introduction

Globally, 2024 was the warmest year on record [1]. In 2023, the second warmest year, people experienced 50 more hot days than would be expected without global warming [2,3]. That year, heat waves caused 47,000 deaths in Europe [2]. As extreme heat events intensify, Germany faces a growing public health burden [3,4]. Between 2010 to 2019, 25,000 additional hospital admissions occurred on the hottest 5% of days compared to moderate days [5]. Heat waves thus pose significant challenges to healthcare systems and professionals, with nurses representing the largest group [6,7]. In addition to the increased workload caused by heat-related patient admissions and care demands, heat waves pose a serious occupational hazard for nurses (e.g. fatigue, headache, dizziness, breathing problems) [[6], [7], [8]].

To address heat-related challenges and nurses’ work stress, inpatient settings must adapt to the intensifying climate crisis. A study by the Ludwig-Maximilian-University (LMU) Medical Center found that hospital nurses feel the least protected from heat compared to those working in nursing homes or outpatient services. They were were also least likely to report the use of heat warnings or heat health action plans (HHAP). Improved institutional adaptation could help reduce stress and safeguard nurses’ health [6]. In Germany, heat protection falls under the responsibility of federal states [9]. In 2017, the German Federal Ministry for the Environment published 'Recommendations for the development of HHAP to protect human health' [9]. In 2023, the German Ministry of Health published the ‘Heat protection plan for health’ [10]. Various other initiatives have also issued recommendations [[11], [12], [13], [14]], covering topics such as patient care adaptation, cooling zones, medication storage, and employee training for extreme heat. However, only a few address the organization of nurses’ work (e.g., longer breaks, which are rarely feasible [7]), despite its key role in hospital resilience [15]. The absence of work organization guidelines may force nurse leaders to act independently, leading to suboptimal work design, excessive workload, reduced quality of patient care, and increased leadership burden. Adapting work organization during a crisis is thus essential to reduce staff strain [[16], [17], [18]].

Germany lacks nationwide efforts in implementation and evaluation of HHAP, both nationally [19] and at the facility level [6,19]. Crisis management in the case of heat events is considered to be poorly developed [[20], [21], [22]]. Interviews with 30 nurse leaders from nine hospitals confirmed this: no hospital had a formalized HHAP, and interventions were fragmented, addressing systemic problems at the individual level [8]. While the findings offered insights and examples, organizational interventions were insufficient, and nursing staff were not involved.

This study aims to extend understanding of organizational interventions that structure nurses’ work, complementing existing HHAPs by involving nursing staff as well as trans- and interdisciplinary perspectives. As heat waves increasingly challenge hospitals, the study also seeks to identify future-relevant interventions. Using a qualitative, scenario-based approach, it addresses three questions:

1. What challenges do heat waves impose on nursing staff in acute care settings?
2. What interventions can be implemented to deal with those heat-related challenges?
3. What other aspects are relevant to hospitals in the context of heat adaptation?

The third question ensures that participant-raised themes beyond the main topics are captured. This open-ended approach leverages the strengths of qualitative research, enabling deeper and more detailed insights.

## 2 Methods

### 2.1 Study design

**Fig. 1:**
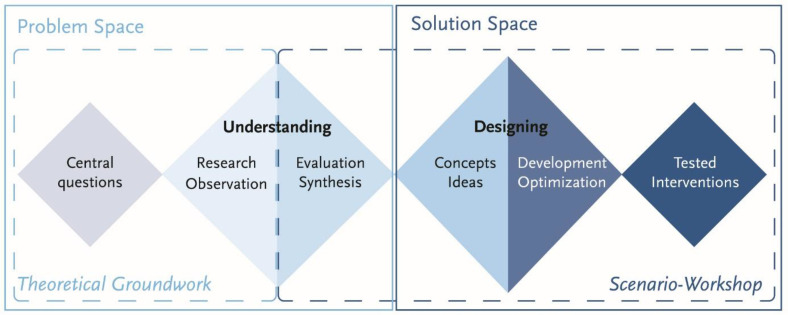
Design thinking process as the basis for workshop structure.

To address the research questions, we conducted a one-day scenario workshop based on the methodology ‘Zukunftswerkstatt’ (Futures Workshop, [23], [24]), incorporating design thinking [25] principles (Fig. 1) and following the criteria for reporting qualitative research (COREQ, 26; Supplementary File 1). This structured approach supports envisioning future scenarios, exploring practical, future-oriented interventions, examining underlying implementation processes, and, more generally, gaining actionable insights relevant to nurses’ work. Participants came from various disciplines within a single hospital. This study followed Consolidated Criteria for Reporting Qualitative Studies. This workshop, which focused on developing interventions to improve hospital responses to heat and nurses’ working conditions, did not constitute human subjects research and was therefore exempt from ethics approval by ethic committee.

### 2.2 Recruitment of participants

Workshop participants were to be employed at the same hospital. Relevant stakeholders from three hospitals were contacted via mail or telephone, with project details shared in an information document. All expressed interest, but one hospital was selected due to its ongoing heat adaptation effort. This specialized trauma and acute care center in a German metropolitan area has 608 beds, 14 departments, and about 2,000 employees (749 nursing professionals). It focuses on treating and rehabilitating patients with severe injuries, burns, and occupational diseases.

After hospital management approved the project, the research and the climate manager teams met to assign tasks. The research team handled conceptualization, external materials, and workshop execution, while the hospital’s climate manager team took responsibility for the recruitment of participants, internal communication, and room organization. Scenario workshops use an inter- and transdisciplinary approach, as complex challenges like the climate crisis require diverse expertise. One of the hospital’s climate managers, also a nurse, recruited participants from multiple professions. The goal was 12 participants; 10 confirmed 8 weeks in advance. On the workshop day, six participants attended; others were absent due to illness, vacation, or internal duties. The final group included two climate managers (a physician and a nurse), a nursing department head, a former nurse now working in hospital management, optimizing nursing service work flow, an occupational health manager, and an employee representative. Though invited multiple times, no more than three nurses were able to participate due to illness or time constraints. All participants had previous collaboration on climate adaptation. All participants gave their informed consent.

### 2.3 Data collection

Three researchers, including two futures research experts, designed the scenario workshop in collaboration with the hospital’s climate manager. We used futures scenarios constructed in advance [27] and followed the ‘Zukunftswerkstatt’ (Futures Workshop) methodology [23,24], enhanced with techniques like the ‘Futures Wheel’ [28], ‘Ideation Canvas’ [29] and an effort–impact matrix [30] (see Table 1 for definitions or Supplementary File 2) to support systematic analysis and encouraged creative problem-solving. The workshop was structured in three phases:

**Table 1:** Definitions of methodology and relevant techniques.

Zukunftswerkstatt / Futures workshop	A participatory method developed by Robert Jungk to enable collective problem analysis, creative visioning, and the development of actionable solutions through three phases: critique, vision, and realization [23].
Design Thinking	A human-centered, iterative approach to innovation that emphasizes empathy, multidisciplinary collaboration, and rapid prototyping to address complex problems and develop user-centric solutions. It integrates understanding, improvement, and application phases to foster creativity and practical outcomes [25].
Futures scenarios	A constructed, plausible narrative of a possible future state, used to explore uncertainties, envisioning and guide strategic decisions [27].
Futures Wheel	A structured brainstorming and visualization method (developed by Jerome C. Glenn) to map out direct, indirect, and cascading consequences of a central trend or event, thereby helping participants anticipate multiple levels of impact and interconnections [28].
Ideation Canvas	A structured tool designed to facilitate creative ideation by guiding individuals or teams through a focused exploration of possible solutions to a defined problem within a set time frame. This approach aims to stimulate innovative thinking and uncover novel solutions [29].
Effort–impact matrix	The Effort-impact matrix is a strategic decision-making tool that helps prioritize tasks by evaluating their potential impact against the effort required for their completion, enabling people to focus on actions that maximize value while minimizing resource expenditure [30].
Silent Discussion	A pedagogical method that prohibits verbal communication, encouraging participants to engage in written exchanges on a given topic. This approach fosters inclusivity by allowing simultaneous participation, reduces anxiety associated with verbal contributions, and promotes critical reflection on the subject matter [31].

Phase 1 – Critique Phase: In this phase, scenarios, prepared in advance, were used to focus and reflect on possible consequences of intensifying heat waves, emphasizing both employee- and facility-related challenges and solutions. The elaborated scenarios were presented stepwise in four escalating levels (see Table 2 or Supplementary File 3). Participants worked in two groups. One group, composed of participants with nursing backgrounds but differing roles (nurse, nurse leader, nurse administrator), focused on the consequences for nurses and their work organization. The second group, consisting of participants with various backgrounds, focused on more general topics regarding the hospital’s workforce. Both groups identified key issues to build a comprehensive understanding of the challenges. For each scenario, participants were asked to propose possible solutions. The results of this activity were handwritten answers from each group for each scenario step. Following the scenarios, participants were asked to each reflect the recent task within the framework of a ‘Silent Discussion’ [31]. The result of this activity was a pinboard with handwritten answers by each participant.

**Table 2:** Scenarios used for the scenario work in Phase 1 (translated by authors).

Initial scenario	*Berlin, August 2030, midday, temperature 35°Celsius in the shade.* The outside temperature has been 35°Celsius in the shade for two days now. There is no sign of the temperature dropping. At the hospital, care for patients who are vulnerable to heat is being adapted. At the same time, the influx of patients due to heat-related illnesses is already increasing. Staff for the non-air-conditioned units are complaining about the workload.
Level 1	The outside temperature has been very high for 5 days now. Since yesterday, it has been 40°Celsius in the shade. The transport infrastructure in the surrounding area is increasingly impaired. Some roads can no longer be used, the main roads are congested, and there are miles of traffic jams, and train services are irregular due to deformed rails. The influx of patients remains high, and the patients on site are increasingly stressed by the persistent heat. The staff is also reaching their limits.
Level 2	The extreme heat continues. In recent days, temperatures have fluctuated between 35° and 40°Celsius in the shade. After more than a week, the transport infrastructure remains impaired. Deliveries are only possible to a limited extent. Stocks are running low. There have already been the first power cuts, which fortunately have been compensated for by emergency generators. There are also initial reports that the drinking water supply is no longer guaranteed in parts of the city. The stress level among employees is rising. There is an increase in sick leave. Patients are frightened.
Level 3	After almost 2 weeks of extremely high outside temperatures, the security of supply is under threat. The power supply is irregular, and emergency generators have to be operated for long periods of time. The supply chains can only guarantee a patchy supply. Sufficient clean drinking water is also not available. There have been a high number of deaths in the last few days, and the cooling capacities are no longer sufficient. The employees who are still able to work are extremely exhausted. Patients have to be turned away again and again.

Phase 2 – Visioning Phase: On the basis of phase 1, ‘What if?’ questions were developed to explore possible futures. The ‘Futures Wheel’ was employed to visualize potential outcomes and opportunities, enabling a structured examination of the implications while encouraging systemic and interconnected thinking and explore possible futures with consideration of the scenarios. Participants place the ‘What if?’ question in the middle of a canvas. They then identify first-order effects (direct impacts) in a surrounding circle, followed by second- and third-order effects (indirect impacts) in subsequent layers. Due to the 1-day time restriction of the workshop, third-order effects were not discussed. Instead, participants discussed possible solutions in the third round. They were instructed to prioritize generating a large number of ideas over refining their quality. Both groups rotated between Futures Wheel canvases to incorporate diverse perspectives. Results of this activity were two Futures Wheels with handwritten ideas (see Supplementary File 4).

Phase 3 – Realization Phase: The final phase centered on formulating actionable interventions. An effort–impact matrix was used to evaluate the feasibility and effectiveness of proposed solutions during the third round of the ‘Futures Wheel’ in Phase 2, ensuring practical and targeted outcomes. In the last step, participants individually developed one solution to the proposed ideas. For that purpose, the ‘Ideation Canvas’ was used, considering relevant stakeholders, timelines, and resources to implement the solution. The result of this activity were handwritten ideation canvases for each participant (see Supplementary File 4).

The workshop took place in the rooms of the hospital and was moderated by one researcher and assisted by two other researchers. Throughout the session, photographic records of the handwritten results of each work step were collected. Additionally, researchers took notes to support photographic records. No audiotapes were made in order to foster an open and creative atmosphere. In addition, the workshop was conducted in multiple group discussions, which would have been technically difficult to record. Photographs of the handwritten participants’ responses (in German) can be found in Supplementary File 4.

### 2.4 Data analysis

As this study employed a qualitative approach, no hypotheses were formulated in order to maintain an open, exploratory perspective, which is a key principle of qualitative research. Data analysis consisted of three main steps and was conducted using MAXQDA 24 and Excel MS software. In the first step, workshop results were discussed in multiple sessions by the workshop researchers and the extended research team (5 researchers in total), using photographic records and researchers’ notes. This resulted in a workshop documentation describing each work step and its outcomes without any abstraction. In the second step, the documentation was sent to participants for feedback. In the final step, one researcher conducted the analysis, integrating workshop documentation, photographic records, researchers’ notes, and participant feedback. Analysis in this step followed qualitative content analysis according to Kuckartz and Rädiker [32], using deductive and inductive codes. The goal of this last step was to condense and abstract the findings, consolidating key challenges, the relevance of heat adaptation, and proposed interventions. The final results are presented in two parts. First, findings are described in detail for each workshop step to enhance transparency. The second part aggregates the results to highlight key challenges, interventions, and relevant topics. To enhance practical applicability, interventions were categorized according to the risk and crisis management cycle [33], a continuous process used by organizations, governments, and healthcare institutions to identify, assess, prepare for, respond to, and recover from potential threats (see also Fig. 4).

## 3 Results

### 3.1 Results by each phase

#### 3.1.1 Critique and visioning phase: analyzing impacts of heat waves

##### 3.1.1.1 Scenario work

In group discussions on the escalating heat scenarios (Table 2), participants identified challenges across the nursing work system, which are part of overall results in Table 3. Interventions were grouped into five themes: interventions referring to 'Nursing staff', 'Patients and provision of care', 'Communication', 'Resources and infrastructure', and the theme 'Other aspects' (e.g., management of the deceased).

**Table 3:** Identified challenges during heat waves for hospitals, nursing staff, as well as patients, and the effects of successful heat adaptation.

**Overall challenges during heat waves**	
*For hospital*	*For nursing staff*
• Increased patient load • Special care for heat-vulnerable individuals is necessary • Patient load in crisis may overwhelm staff capacities • Increased material consumption • Scarce resources: Time, staff, material, finances • Allocation of resources • Distribution of goods • Heat adaptation as leadership-level priority: Management commitment is needed (especially middle management as a critical leverage point) • Balancing needed staffing with labor regulations • Integration of heat adaptation into hospital planning (e.g., finance planning) and governance • Continuous evaluation of heat health action plan	• Increased workload without adaptation • Decreased concentration • Aggression potential • Burden on health and reduction of nurses’ stress during and after the heat event • Emotional strain: fear, feelings of helplessness, psychological avoidance • Reduced motivation for work • Gaps in understanding of heat-related health risks
	*For patients*
	• Balancing patient care demands with the health and well-being of staff • Risk of accidents, medical malpractice, due to decreased care quality, and decreased concentration
**Challenges when heat wave is intensifying**	
• Supply challenges due to issues in the provision of power, hygiene materials, food, and water: ○ affects the internal organization and logistics of hospital supplies ○ affects the quality of patient care and increases nurses’ burden • Access difficulties for nursing staff caused by: ○ Irregularities in public transportation ○ Inaccessibility of roads • Communication barriers between internal and external staff: ○ Important information is often stored only in internal systems ○ External employees have limited access to the necessary data • Risk of epidemics (e.g., due to contaminated food or water)	
**Effects of successful heat adaptation**	
• Better allocation of resources and more efficient care • Increase in scarce staff resources due to a decrease in absence and increased staff satisfaction • Heat health action plan would free up the capacities of middle management • Increased care quality and patient safety • Increased staff satisfaction • Decrease in psychological burden • Staff attachment to the hospital as an employer • Validation of nursing staff by management • Decrease in feelings of overexertion and performance pressure • Loyalty and solidarity within the team and better teamwork	

###### 3.1.1.1.1 Nursing staff

With regard to interventions related to the theme 'Nursing staff', participants discussed adaptation of duty rosters (e.g., increasing staff per shift, adjusting work hours), consideration of the deployment of vulnerable staff, organization of breaks (e.g., longer or more short breaks) and break rooms (e.g., cooling, provision of drinks), adaptation or reduction of work tasks, and adequate clothing (e.g., lighter clothes). With increasing crisis severity, interventions extended to providing food and accommodation, relocating work (where feasible), and offering psychological support (e.g., crisis intervention teams).

###### 3.1.1.1.2 Patients and provision of care

In the theme ‘Patients and provision of care’, interventions such as lighter dishes and organization of room occupancy (e.g., more heat-vulnerable patients are transferred to cooler rooms) were mentioned. To efficiently manage staff, work tasks, and patient transfer, the core admission time of patients with heat stress needs to be considered. With an escalating crisis, following care task priority concepts and transferring patients to other health facilities are relevant. Finally, staff need to ensure at least basic patient care.

###### 3.1.1.1.3 Communication

Regarding ‘Communication’, informing employees about risks and repeating heat-related training becomes relevant at the beginning of a heat event. As the situation escalates, ensuring internal communication (e.g., via mobile apps), coordination with administrative units (e.g., emergency staffing and services), and the hospital’s crisis unit becomes essential. Communication to the general population about helpful behavior is also seen as an option.

###### 3.1.1.1.4 Resources and infrastructure

The majority of the mentioned interventions can be found in the theme ‘Resources and infrastructure’. At the beginning of the heat event scenario, more material resources such as cooling devices and drinks are provided. With an ongoing crisis situation, interventions focus more on saving and securing material resources (e.g., rationing of water, material, and stockpiling). Organizing internal supply structures and securing the external supply chain becomes increasingly important (e.g., prior cooperation with delivery services prioritizing hospitals). Staffing and care capacity must be expanded (e.g., use of temporary staff, limiting admissions to emergency cases, closure of rooms or units). If the situation continues to escalate, staff, material, time, and care capacity resources need to be secured. Scarce staff resources are optimally organized by mutual support across units. Unnecessary devices need to be switched off. Only basic and necessary tasks need to be performed. Patients need to be discharged as soon as possible. In general, staff mobility to the hospital needs to be ensured (e.g., provision of bikes, cooperation with public transportation). Not only the operational but also the administrative staff needs to be considered.

###### 3.1.1.1.5 Other aspects

Other aspects that are considered by participants are the cooling of indoor temperature by shading and ventilation, the provision of cooling zones for staff and patients, and the safe storage of medication. At the highest level of escalation, the availability of cooled rooms for deceased individuals was considered important.

##### 3.1.1.2 Silent discussion and futures wheel

**Fig. 2:**
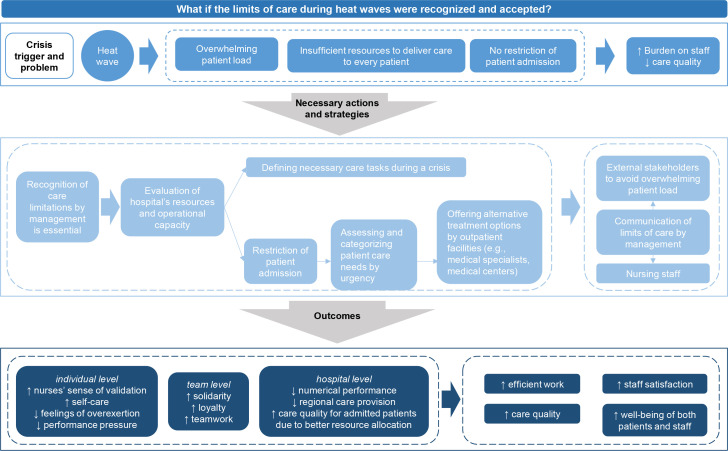
Flow chart of participant’s answers on the Futures Wheel question ‘What if the limits of care during heat waves were recognized and accepted?’.

**Fig. 3:**
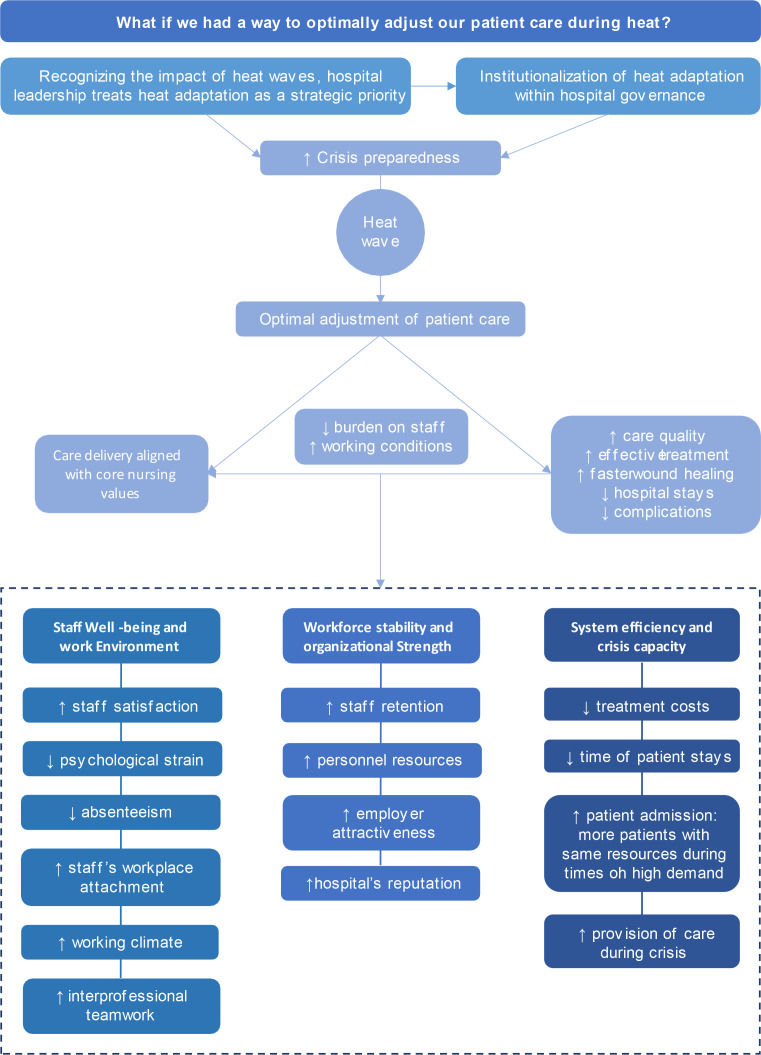
Flow chart of particpant’s answers on the Futures Wheel question ‘What if we had a way to optimally adjust our patient care during heat?’.

In the following ‘Silent Discussion’, workshop participants emphasized the critical need for a crisis management team responsible for preparedness, communication, and implementing protective measures. Participants also highlighted the importance of increased financial resources, improved staff motivation, and enhanced health literacy, both among patients and, in some cases, among non-medical staff. Finally, participants stressed the importance of balancing patient care demands with the health and well-being of hospital staff and their emotional strain. Based on the ‘Silent Discussion’, participants chose two ‘What if?’ questions to continue working with the ‘Futures Wheel’. The results of each futures wheel are summarized in a flow chart shown in Figs. 2 and 3..

#### 3.1.2 Realization phase: intervention development

At the end of the visioning phase, participants developed interventions using the Futures Wheel exercise. During the third phase (realization), these interventions were organized in a plenary session and assessed using an effort-impact matrix. In the next step, six interventions were selected and further refined by the participants using the Ideation canvas. The three processes revealed that many interventions represented partial steps toward a broader, more comprehensive heat adaptation strategy. Participants see heat adaptation as a leadership-level priority and recognize the need to incorporate heat adaptation officially into the hospital’s strategic planning and structures, particularly into economic planning. This includes the need for additional personnel resources to conceptualize and evaluate interventions, as well as the proactive allocation of financial resources to support staffing and technical solutions. Interventions developed during realization phase were grouped into three overarching categories:

1. Elaboration of a heat health action plan and corresponding guidelines,
2. Development of modular, obligatory training programs,
3. Introduction of a digital application for every employee’s mobile phone.

##### 3.1.2.1 Elaboration of a heat health action plan and corresponding guidelines

The implementation of a facility-wide HHAP emerged as a central tool to aggregate interventions, communicate them, and gain hospital-wide awareness. The comprehensive, hospital-wide HHAP will be developed and continuously updated after every heat event.

The integration and conceptualization of guidelines into the overall plan is seen as a key element to give staff clear instructions, directives, and recommendations. This approach is expected to enhance confidence in nursing tasks, standardize procedures, increase efficiency, and reduce errors. Existing guidelines need to be revised in view of heat adaptation, while new guides will be created for various departments and heat warning levels, with corresponding area and task prioritizations. The definition of prioritized areas and nursing tasks is important to balance scarce resources during heat waves. The prioritized tasks and areas, as well as the guides, need to be transparently communicated to all hospital employees.

Finally, communication of the standards and the HHAP is identified as a key element. In the past, nursing middle management invested a lot of working time in informing nursing staff about interventions and strategies. Clear and transparent communication before and during heat waves would free up the capacity of middle management to follow other important tasks. According to participants, to achieve this, communication needs to be organized top-down with the deployment of multipliers to ensure heat adaptation knowledge in various hierarchical levels. Practical trainees and middle managers were identified as key communication nodes for disseminating information and motivating nursing staff. Furthermore, external communication is considered crucial as regional stakeholders (ambulances, other hospitals, long-term care facilities, and political decision-makers, etc.) are informed about the hospital’s heat wave strategies and can adapt accordingly.

##### 3.1.2.2 Development of modular, obligatory training programs

Another intervention to ensure heat adaptation knowledge, communication, compliance, and practical use is the development of modular, obligatory training programs, such as basic and role-specific heat preparedness courses, which will be repeated annually. Training content includes personal heat protection measures, health impacts, practical interventions, and overarching strategies. Different heat scenarios can be used to support understanding and illustrate the impacts of heat events. The program will be held in person, but a version for self-study needs to be created to reach every employee. It is also essential to include staff from subsidiary companies in the training process.

##### 3.1.2.3 Introduction of a digital application for every employee’s mobile phone

To further support and ensure real-time communication, even in crisis situations, the introduction of a digital application for every employee’s mobile phone is suggested. This obligatory but free-of-charge staff app will serve as a central communication tool, providing real-time access to crisis information, heat-related protocols, duty rosters, team meetings, peer-to-peer communication, and training materials at any time.

### 3.2 Aggregation of results

**Fig. 4:**
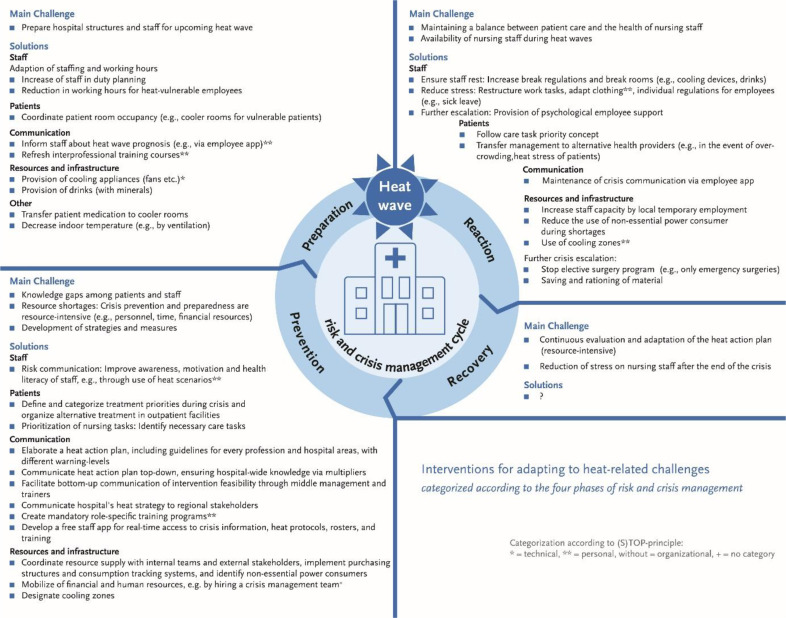
Summary of the main technical, organizational, and personal interventions to adapt to heat-related challenges, developed by participants; a comprehensive list is provided in Supplementary File 5.

All challenges and anticipated effects of successful heat adaptation are summarized in Table 3 (Question 1). All proposed interventions, assigned to the four phases of crisis management (prevention, preparation, response, and recovery), are summarized in detail in Supplementary File 5. An overview of key interventions is provided in Fig. 4. Almost all of the found interventions can be categorized as organizational interventions, according to the (S)TOP-principle, a framework for occupational safety that prioritizes Substitution to Technical measures, and prefers them to Organizational measures, and Personal protective strategies (Question 2). During further analysis of the results, four themes emerged: resource intensity of heat adaptation; challenges without solutions; difficulties in developing interventions; and ethical and moral issues of heat adaptation (Question 3). The first theme 'rBackspaceResource intensity of heat adaptation' was a recurring topic during the workshop. According to participants, effective crisis management requires additional staff, materials, financing, and time. Regarding the second theme, it became evident that multiple challenges were not completely analyzed during the workshop. For example, one important topic throughout the workshop was that heat adaptation is a management priority, with middle management being a key leverage point, and needs to be integrated into planning structures and governance. However, concrete ideas to gain institutional support were lacking. One idea was to use internal communication tools to highlight health and structural impacts. Another way to promote awareness is through climate-related events (e.g., greening hospital grounds). Other challenges without solution are the reduction of nurses’ stress as well as the evaluation process of heat adaptation strategies and interventions after the end of the crisis. Moreover, no interventions were mentioned for the recovery phase. Although operational topics such as staffing and nursing practice were discussed, they were not central to the interventions developed. For example, the idea of 24-h shifts during heat waves to allow for longer rest periods was discussed but ultimately deemed unfeasible due to labor regulations. Participants also observed that the number and detail of interventions declined with increasing scenario escalation. These are examples for the third theme 'Difficulties in develping interventions'. Finally, the regulation of patient admission raises ethical and moral questions (i.e., triage) that need to be discussed ahead of the crisis.

## 4 Discussion

We conducted a scenario workshop to identify work-organizational interventions hospitals can use to reduce nurses' heat-related stress, addressing gaps in previous research that overlooked nurses' perspectives and lacked future-oriented planning amid the intensifying climate crisis. The participatory, scenario-based qualitative approach proved valuable. Throughout the workshop, participants engaged in in-depth discussions about how to address heat-related challenges within the hospital. The complexity of the issue and the level of effort required for adequate preparedness, focusing on present and future challenges, became increasingly evident. In the following, we discuss the results in line with the research questions.

### 4.1 Q1 challenges

Participants identified heat waves as causing resource scarcity across staffing, finances, time, and material shortages, which are similar to findings regarding the management of the COVID-19 pandemic [34] and are characteristic of crisis situations. Challenges mentioned in this study are similar to challenges reported by other studies (e.g., health problems, such as fatigue and concentration problems, increased patient load, and higher care demands) [[6], [7], [8]]. Future heat waves pose additional risks, including disruptions in power, food, water, and hygiene supply chains, as well as limited access of staff to the facility due to infrastructural hindrances (e.g., no public transportation, roads destroyed by heat) and communication barriers between internal and external staff. Resource scarcity, limited hospital access and potential communication barriers need to be integrated into formal heat strategies. During scenario work, resource consumption spiked early in the crisis to meet immediate needs. As conditions worsened, essential supplies like water and hygiene materials required rationing. This underscores the critical importance of anticipating resource scarcity and planning with a forward-looking, systemic approach to heat adaptation. Beyond the physical strain of heat, the emotional and psychological impact on nursing staff mentioned during Silent Discussion—such as fear, feelings of helplessness, and psychological avoidance—calls for deeper exploration and the integration of targeted mental health strategies into heat preparedness plans. This may involve structured peer-support groups, access to counseling services during heat emergencies, and regular resilience training for staff.

### 4.2 Q2 interventions

Participants identified a broad variety of interventions to tackle the challenges stated above. A key insight was the essential role of a facility-wide HHAP. There is evidence that formalized plans, including heat alerts, are not widely implemented in hospitals across Germany [6,8,19]. Clear action guidelines and heat adaptation strategies must be communicated to staff, with middle management helping bridge strategy and operations. Identified interventions can support the elaboration of practice-oriented plans.

Interventions can be categorized according to the (S)TOP-principle and allocated to the four phases of risk and crisis management. Zink et al. [8] identified technical, organizational, and personal interventions for adapting to heat in hospitals and long-term care settings, with the majority interventions reported by hospital staff being personal interventions. In contrast, most interventions identified in this study were organizational, reflecting a shift toward adapting hospital systems rather than addressing nurses’ burdens individually. Moreover, interventions identified in the study by Zink et al. [8] focus on the mitigation of physiological impacts (e.g., interventions of cooling the rooms through shading and ventilation or the human body through foot baths). Interventions presented here go beyond physiological impacts and concentrate on impacts on hospital processes (e.g., communication, staff management, use of resources, adaptation of infrastructure). As these interventions stem from real-world field experience, they contribute valuable, practice-oriented insights mentioned by other studies [6,7,35] and should be given particular consideration in recommendations of heat adaptation. While there was significant focus on internal processes, cross-sector collaboration with external stakeholders, such as public health agencies, local governments, long-term care facilities, or emergency services, should be further emphasized, as it plays a crucial role in crisis response, as seen during the COVID-19 pandemic [34,36].

### 4.3 Q3 other aspects

Additional key takeaways on heat adaptation emphasize the need for stronger internal awareness building and institutional support, especially from leadership and middle management. Successful implementation of suggested interventions and HHAPs involves resource commitment (e.g., recruiting a crisis management team), internal communication structures, ongoing heat adaptation evaluation, staff training, and digital tools (e.g., employee app as suggested in this workshop). Different sources for training programs for nursing staff are already available, focusing on geriatric nursing [37,38] .

#### 4.3.1 Evaluation of HHAPs

Evaluation is resource-intensive and was not addressed specifically during the workshop. There are examples of successful evaluations in care settings [39], with helpful material available (e.g., in German, [38]). Regular post-heat wave debriefings with staff, patient (advocacy), and management could provide insights into the successes and shortcomings of the interventions implemented during the heat wave. This feedback loop would ensure that heat adaptation becomes a dynamic, evolving process, rather than a static plan.

#### 4.3.2 Institutional support

Institutional support and the development of a hospital culture that genuinely prioritizes heat preparedness and values sustainable practices are essential. Organizational psychology suggests several mechanisms to promote bottom-up change. Proactive behaviors, such as voicing constructive suggestions for heat adaptation, can play a critical role in drawing leadership attention to emerging needs (Voice Behavior, 40). In parallel, the concept of 'job crafting' [41] highlights how employees can reshape their tasks and responsibilities to align more closely with heat adaptation goals, thereby demonstrating initiative and commitment. Building trust-based relationships with leadership further increases the likelihood that leadership will take these suggestions seriously (Leader–Member Exchange theory, 42). Moreover, communication strategies that emphasize strengths and positive examples*—*drawing from the Appreciative Inquiry approach [43]*—*can foster constructive dialogue and reduce resistance to change.

In the context of heat adaptation, employees can play a key role in raising awareness and generating institutional support by strategically emphasizing specific aspects. These include promoting health literacy among staff, administration, and leadership, highlighting the hospital’s legal obligations regarding occupational health and patient care (e.g., ArbSchG, 44, heat protection plan for health by the Ministry of Health, [10]), and pointing out the cost-effectiveness of preventive measures. Such efforts can be integrated through staff training, discussion in meetings, or small-scale initiatives. Additionally, employees can underscore the reputational benefits and improved staff retention associated with proactive heat adaptation. Notably, employee motivation is a critical factor in the successful implementation of HHAPs [45].

#### 4.3.3 Recovery of nursing staff after a heat event

After experiences during the COVID-19 pandemic, an S3 guideline - Germany’s highest standard of clinical practice guidelines - ‘Mental health of healthcare workers in protracted crises and disasters’ has been developed [46], but interventions implemented during the recovery phase of a crisis are equally important and were described less frequently after the COVID-19 pandemic [34]. In this study the importance of supporting nursing staff recovery after heat events was emphasized, but the development of specific interventions was limited due to time constraints. For example, feedback from nurses after a heat event could be helpful in identifying suitable and individual interventions for physical and mental recreation. On hospital-level, potential measures include peer-support-programs, supervision and debriefings, flexible working hours, or task-specific role adjustments. On individual-level, suggestions include practicing stress management techniques, taking regular breaks, and recognizing health issues and seeking support early. On the health system level, measures such as establishing national occupational safety standards, funding mental health and resilience programs, coordinating workforce planning across regions, and issuing evidence-based guidelines can support the recovery and well-being of nursing staff.

#### 4.3.4 Difficulties in intervention development

Several difficulties in designing suitable interventions became apparent through the workshop results. First, while elaborating overarching strategy, practical, staff-centered interventions were less common or often remained superficial. These require further development and contextualization. One possible explanation may be that certain operational details only become evident during an actual crisis. Realistic crisis simulations involving all relevant personnel may therefore be more effective in identifying specific, actionable interventions at the unit level. Another example of a realistic simulation is LÜKEX 26, a cross-state and interdepartmental crisis management exercise in Germany, focusing on the management of drought and heat, while taking cascading effects into account [47].

Second, as the heat-scenario escalated, participants proposed fewer interventions. This suggests that participants may have found it difficult to envision extreme crisis conditions, particularly when projected in the future. This observation highlights the value of scenario-based foresight work in fostering future-oriented thinking and strengthening crisis preparedness. For both difficulties, the involvement of experts in crisis management or foresight methods could support hospitals’ efforts to prepare for extreme heat events.

#### 4.3.5 Ethical and moral questions

During the visioning phase, participants raised ethical and moral questions regarding which patients, and under what conditions, can be treated during an escalating crisis. Recognizing the need to prioritize certain patients over others can cause nurses moral distress. Ethical frameworks and decision-making guidelines could be developed to help healthcare providers navigate the challenges of limited resources during extreme heat events. Ensuring that these guidelines are part of the HHAP would help alleviate moral dilemmas during a crisis.

### 4.4 Limitations and strengths

Limitations of the study include the small sample size (six participants) and the fact that the workshop was held at only one hospital. For these reasons and because of the qualitative design, the findings lack generalizability and may not fully represent the experiences or needs of other hospitals. Another limitation is the missing involvement of more medical and non-medical professions that play an important role in patient care. Nevertheless, the results offer an in-depth perspective with focus on the future developed by an interdisciplinary team with practical experience which is considered a major strength of our design compared to other quantitative and qualitative designs.

### 4.5 Practical implications

The study’s findings offer practical guidance for hospital management’s heat adaptation efforts. Implementing a HHAP*—*with attention to both staff and patient well-being*,* strengthening internal and external communication and cooperation, preparing staff by creating risk awareness, and ensuring staff training*—*will help to reduce the negative impacts of heat waves. Heat adaptation should be integrated into hospital governance (e.g., in hospital’s emergency planning, leadership prioritization) and resource planning to create a more resilient hospital. Furthermore, hospitals must answer ethical and moral questions regarding patient prioritization due to resource scarcity during a crisis. These must be answered ahead of a crisis and are subject to broader discussion in society. Guidelines may help to communicate the hospital’s position towards nursing staff.

The level of detail and the variety of interventions identified in this study and the previous study by the same authors [8] demonstrate the complexity and facility-specificity of heat adaptation. Therefore, it is recommended that hospitals create resources to elaborate on interventions tailored to a hospital’s layout, patient population, staff needs and workflow and adapt accordingly. In this process, it is important to develop short-, medium-, and long-term interventions during all four phases of crisis prevention. It is also recommended that multiple warning levels be integrated to differentiate between crisis intensities. Participatory foresight methods and realistic crisis simulations can support effective planning, improve crisis response, and enhance staff motivation and engagement.

Additionally, exchange between hospitals can generate best practices. The Klinikum LMU initiative for the geriatric care sector [48] is an example of such an approach. Collaboration with external stakeholders can be a promoting factor during the crisis [34]. Regional networks could be involved prior to a crisis to increase the hospital’s resilience [36]. For example, hospitals’ resilience can be supported by public programs to raise awareness and prevent heat-related health issues in the community. Moreover, external stakeholders can adapt findings of this study to improve their heat resilience. Public health authorities could collaborate with community organizations to distribute cooling devices or hydration supplies. Primary care providers might proactively identify high-risk patients and coordinate follow-up during heat events. Home care services could encourage team-based flexibility so staff can swap or adjust visits to reduce individual heat burden. At the national level, health systems could promote cross-sector coordination between healthcare, social services, and urban planning to strengthen systemic preparedness.Finally, including climate resilience into hospitals' resilience strategy is essential in the context of confrontation with multiple crises (e.g., the COVID-19 pandemic, the climate crisis, and Russia’s invasion of Ukraine). Climate-related hospital admissions already account for an estimated €174 billion annually [49]. As part of Germany’s critical infrastructure [50], hospitals need financial support from the federal state or government to implement suggested interventions and to guarantee continuity of care during a crisis. In 2023, German hospitals had a financial deficit of 9 billion euros. Public funding only covered 50% of the necessary innovation costs [51].

### 4.6 Implications for research

The study highlights the need for further research into heat adaptation strategies, particularly with a focus on operational interventions at the unit level, the role of middle management, effective ways to gain institutional support and raise climate change awareness, and how to continually evaluate the effectiveness of HHAPs within the hospital. Research on the effectiveness of interventions during a crisis is difficult and rare [52]. Nevertheless, it is essential to assess the impacts of proposed interventions on nurses’ health and work-related stress. There are hints that interventions leading to overall successful crisis management can have negative effects on staff [53]. Furthermore, many initiatives and intervention recommendations exist to tackle heat-related challenges. However, evidence indicates that these are not considered in hospitals. Further research is needed to explore how knowledge is translated into practice and to identify organizational, cultural, and structural barriers to heat adaptation.

Finally, with increasing crisis escalation during scenario work, identified interventions decreased. This pattern highlights the difficulty of envisioning distant or extreme future conditions and reinforces the relevance of foresight-based approaches. Hence, further research should consider focusing on foresight methods to prepare for future crises successfully. Broader insights may also be gained by involving a wider range of disciplines and nursing staff, increasing sample size in general and conducting the workshop in multiple hospitals with different specializations. Furthermore, extending the workshop duration could also help to develop more detailed and operationally focused interventions, particularly at the unit level.

## 5 Conclusion

This study highlights the complexity of preparing hospitals—and particularly nursing staff—for intensifying heat-related challenges. Through a participatory scenario-based workshop, we identified a wide range of organizational interventions rooted in practical experience. While many of the proposed interventions focused on internal processes, the findings emphasize the urgent need for comprehensive, facility-specific Heat Health Action Plans that integrate organizational, procedural, and psychological components of crisis preparedness. A shift from individual burden to structural solutions was evident, underscoring the importance of systemic planning over isolated mitigation strategies.

The findings highlight challenges in developing interventions, including a lack of operational detail and difficulty envisioning extreme crises. This underscores the need for future thinking and crisis simulations, supported by expertise in foresight and crisis management. Additionally, the study underscores the role of staff-led initiatives in raising awareness and promoting internal engagement. Institutional commitment and interdisciplinary collaboration—especially with external stakeholders—emerge as critical for successful adaptation.

Despite its limited sample size, the study offers a nuanced, practice-oriented contribution to the development of heat adaptation strategies. It also identifies underexplored areas such as post-crisis recovery, evaluation practices, and the moral implications of patient prioritization during resource scarcity. These findings have implications for both policy and practice: hospitals must embed climate resilience into governance structures, resource allocation, and ethical frameworks.

## CRediT authorship contribution statement

**Maria Zink:** Writing – review & editing, Writing – original draft, Visualization, Resources, Project administration, Methodology, Investigation, Formal analysis, Data curation, Conceptualization. **Andrea Nakoinz:** Writing – review & editing, Resources, Investigation. **Ulrike Krol:** Writing – review & editing, Resources, Investigation, Conceptualization. **Niels Jansen:** Writing – review & editing, Resources, Methodology, Investigation, Formal analysis, Conceptualization. **Franziska Jung:** Writing – review & editing, Supervision, Conceptualization. **Steffi G. Riedel-Heller:** Writing – review & editing, Supervision. **Katharina M.A. Gabriel:** Writing – review & editing, Supervision.

## Declaration of competing interest

None.

## Appendix Supplementary materials

Image, application 1

Image, image 2 dummy alt text

Image, application 3

Image, application 4

Image, application 5

## Footnotes

Supplementary material associated with this article can be found, in the online version, at doi:10.1016/j.joclim.2026.100659.
