# Supplementary figures and images for: Heat-related challenges and interventions in hospitals: A future-oriented, qualitative approach to improve nurses' working conditions
Source: J Clim Chang Health. 2026 Apr 10;28:100659. doi: 10.1016/j.joclim.2026.100659 (PMC13091379; doi:10.1016/j.joclim.2026.100659)

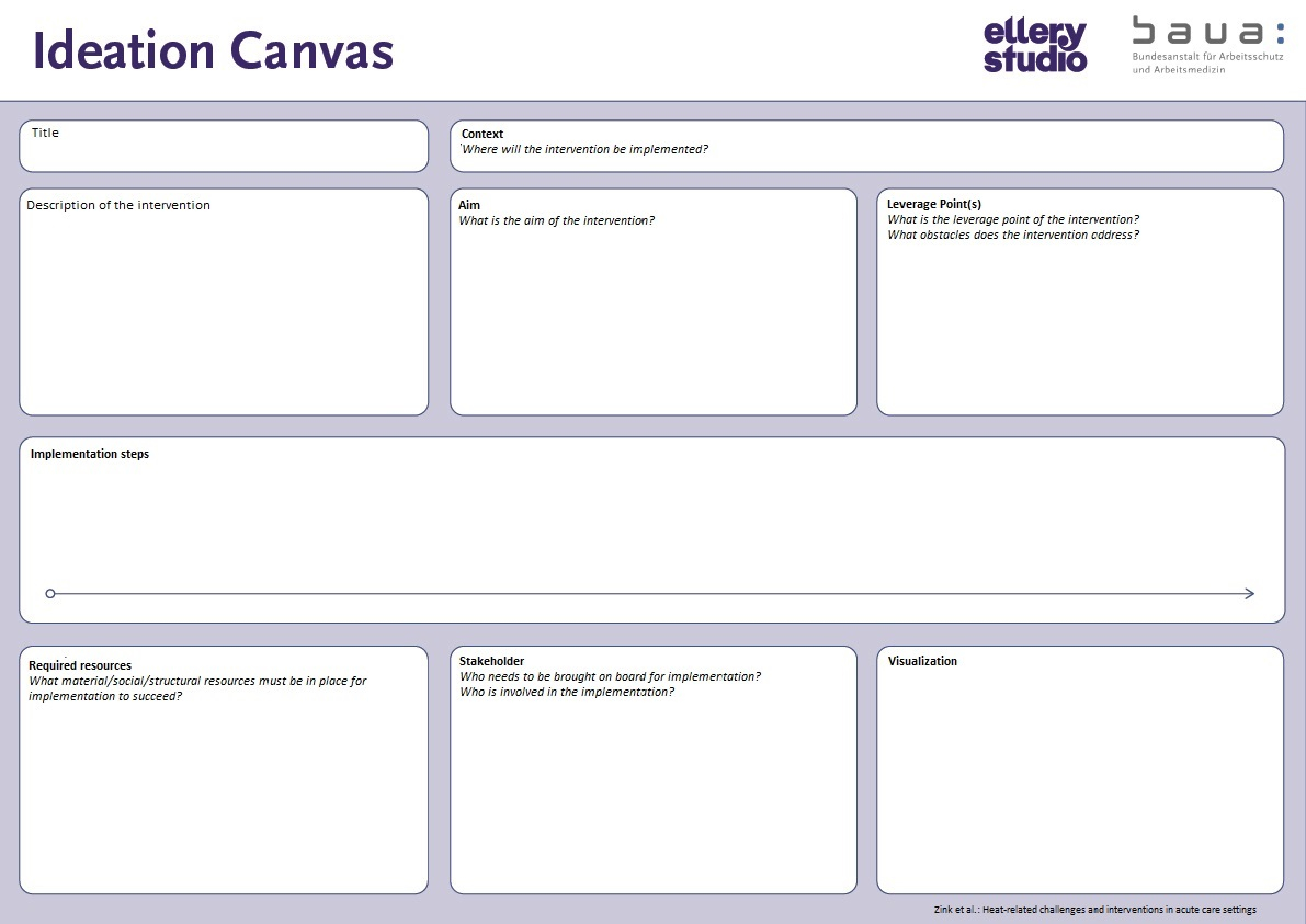

Supplement: Supplementary file 2 [file mmc2.jpg]
