# Supplementary material for: Heat-related challenges and interventions in hospitals: A future-oriented, qualitative approach to improve nurses' working conditions
Source: J Clim Chang Health. 2026 Apr 10;28:100659. doi: 10.1016/j.joclim.2026.100659 (PMC13091379; doi:10.1016/j.joclim.2026.100659)
Supplement: Supplementary file 3 [file mmc3.pdf]

Supplementary File 3

Simulation with scenarios (English Version)

**Introduction Simulation**	**5"**	Allocation to the three groups<br><br>1st card: Each group is given a task description. This may or may not overlap completely with your own area of responsibility. Therefore, think specifically about your own area of responsibility, but also try to adopt perspectives from other relevant areas.<br><br>1st scenario card: You will be given a card with a short scenario. Then you should answer the following question ... first on your own, then in your group (questions on sheet of paper or throw on).	
Map: Information for the group / task description		**Patient care team**	**Employee management team**
		You are responsible for patient care. You must consider all activities that are necessary for the patient's well-being. Don't forget that you have to keep an eye on your own workload.	You are responsible for the organization of patient care and personnel management. Your aim is to ensure patient care and at the same time reduce the workload of the staff, especially the nurses.
	5"	Read through; ask questions	
Start scenario for all; no variation across teams	**15"**	**Berlin, August 2030, midday, temperature 35° Celsius in the shade.**<br><br>The outside temperature has been 35° Celsius in the shade for two days now. There is no sign of the temperature dropping. At ukb, care for patients who are vulnerable to heat is being adapted. At the same time, the influx of patients due to heat-related illnesses is already increasing. Staff on the non-air-conditioned wards are complaining about the workload.	
Task		1. what does this mean for your area of responsibility?<br><br>2. how are you dealing with the situation?	

Level 1:<br><br>no variation across teams	10"	The outside temperature has been very high for five days now. Since yesterday it has been 40° Celsius in the shade.<br><br>The transport infrastructure in the surrounding area is increasingly impaired. Some roads can no longer be used, the main roads are congested and there are miles of traffic jams, and train services are irregular due to deformed rails.<br><br>The influx of patients remains high and the patients on site are increasingly stressed by the persistent heat. The staff are also reaching their limits.
Task		1. what does this mean for your area of responsibility?<br><br>2. how are you dealing with the situation?
Level 2:<br><br>No variation across teams	10"	The extreme heat continues. In recent days, temperatures have fluctuated between 35° and 40° Celsius in the shade.<br><br>After more than a week, the transport infrastructure remains impaired. Deliveries are only possible to a limited extent. Stocks are running low. There have already been the first power cuts, which fortunately have been compensated for by emergency generators.<br><br>There are also initial reports that the drinking water supply is no longer guaranteed in parts of the city.<br><br>The stress level among employees is rising. There is an increase in sick leave. Patients are frightened.
Task		1. what does this mean for your area of responsibility?<br><br>2. how are you dealing with the situation?
Back-up scenario, if time:<br><br>Level 3: no variation across teams		After almost two weeks of extremely high outside temperatures, the security of supply is under threat.<br><br>The power supply is irregular and emergency generators have to be operated for long periods of time. The supply chains can only guarantee a patchy supply. Sufficient clean drinking water is also not available.<br><br>There have been a high number of deaths in the last few days and the cooling capacities are no longer sufficient.<br><br>The employees who are still able to work are extremely exhausted. Patients have to be turned away again and again.
Task		1. what does this mean for your area of responsibility?<br><br>2. how are you dealing with the situation?

Simulation mit Szenarien (Deutsche Version)

**Einleitung Simulation**	**5“**	Zuteilung zu den drei Gruppen<br><br>1. Karte: Jede Gruppe bekommt eine Aufgabenbeschreibung. Diese kann, muss sich aber nicht vollständig mit dem eigenen Aufgabenbereich überschneiden. Denkt daher zwar konkret an Euren eigenen Aufgabenbereich, versucht aber auch Perspektiven anderer relevanter Bereiche zu übernehmen.<br><br>1. Szenario-Karte: Ihr werdet eine Karte mit einem kurzen Szenario bekommen. Dann sollt ihr folgende Frage .. zunächst alleine, dann in eurer Gruppe beantworten (Fragen auf Blatt oder anwerfen).	
Karte: Informationen für die Gruppe /Aufgabenbeschreibung		**Team Patientinnenversorgung**	**Team Mitarbeitendenmanagement**
		Ihr seid für die Versorgung der Patientinnen verantwortlich. Ihr müsst alle Tätigkeiten bedenken, die für das Patientenwohl nötig sind. Vergesst dabei nicht, dass ihr dabei eure eigene Belastungsgrenze im Blick habt.	Ihr seid für die Organisation der Patientinnenversorgung sowie das Personalmanagement zuständig. Euer Ziel ist, die Patientinnenversorgung sicherzustellen und gleichzeitig die Belastung des Personals, vor allem der Schwestern und Pfleger, zu reduzieren.
	5“	Durchlesen; Fragenstellen	
Start Szenario für alle; keine Variation über Teams hinweg	**15“**	**Berlin, August 2030, Mittagszeit, Temperatur 35° Celsius im Schatten.**<br><br>Seit zwei Tagen beträgt die Außentemperatur 35° Celsius im Schatten. Eine Temperatursenkung zeichnet sich nicht ab. Im ukb wird die Versorgung hitzevulnerabler Patient:innen angepasst. Gleichzeitig steigen bereits die Patient:innenzuströme aufgrund hitzebedingter Erkrankungen. Das Personal auf den nicht-klimatisierten Stationen klagen über die Arbeitsbelastung.	
Aufgabe		1. Was bedeutet das für euren Zuständigkeitsbereich?<br><br>2. Wie geht ihr mit der Situation um?	

Level 1: keine Variation über Teams hinweg	10“	Seit nun fünf Tagen ist die Außentemperatur sehr hoch. Seit gestern beträgt sie 40° Celsius im Schatten.<br><br>Die Verkehrsinfrastruktur im Umkreis ist zunehmend beeinträchtigt. Straßen können teilweise nicht mehr genutzt werden, die Hauptverkehrsstraßen sind überfüllt und es gibt kilometerlange Staus, der Zugverkehr ist aufgrund verformter Schienen unregelmäßig.<br><br>Der Patientinnenzustrom bleibt erhöht und die Patientinnen vor Ort sind zunehmend durch die anhaltende Hitze belastet. Auch das Personal kommt an die Belastungsgrenze.
Aufgabe		1. Was bedeutet das für euren Zuständigkeitsbereich?<br><br>2. Wie geht ihr mit der Situation um?
Level 2:<br><br>keine Variation über Teams hinweg	10“	Die extreme Hitze dauert weiter an. Die Temperaturen schwanken in den letzten Tagen zwischen 35° und 40° Celsius im Schatten.<br><br>Nach über einer Woche bleibt die Verkehrsinfrastruktur beeinträchtigt. Zulieferungen sind nur eingeschränkt möglich. Die Lagervorräte gehen zur Neige. Es kam bereits zu ersten Stromausfällen, die zum Glück durch Notstromaggregate ausgeglichen werden konnten.<br><br>Außerdem gibt es erste Berichte, dass die Trinkwasserversorgung in Teilen der Stadt nicht mehr gewährleistet ist.<br><br>Das Stresslevel bei den Mitarbeitenden steigt. Es kommt vermehrt zu Krankmeldungen. Patientinnen sind verängstigt.
Aufgabe		1. Was bedeutet das für euren Zuständigkeitsbereich?<br><br>2. Wie geht ihr mit der Situation um?
Back-Up Szenario, falls Zeit:<br><br>Level 3: keine Variation über Teams hinweg		Nach fast zwei Wochen anhaltenden extrem hoher Außentemperaturen ist die Sicherstellung der Versorgung bedroht.<br><br>Die Stromversorgung ist unregelmäßig sichergestellt und Notstromaggregate müssen über längere Zeit betrieben werden. Die Lieferketten können nur eine lückenhafte Versorgung gewährleisten. Auch genügend sauberes Trinkwasser steht nicht zur Verfügung.<br><br>Es kam in den letzten Tagen zu einer hohen Anzahl von Todesfällen und die Kühlkapazitäten reichen nicht mehr aus.

		Die noch arbeitsfähigen Mitarbeitenden sind extrem erschöpft. Es müssen immer wieder Patient:innen abgewiesen werden.
Aufgabe		1. Was bedeutet das für euren Zuständigkeitsbereich?<br><br>2. Wie geht ihr mit der Situation um?
