# Supplementary material for: Heat-related challenges and interventions in hospitals: A future-oriented, qualitative approach to improve nurses' working conditions
Source: J Clim Chang Health. 2026 Apr 10;28:100659. doi: 10.1016/j.joclim.2026.100659 (PMC13091379; doi:10.1016/j.joclim.2026.100659)

Supplementary File 4

Examples of participants' responses

Example of Futures Wheel

What if the limits of care during heat waves were recognized and accepted?

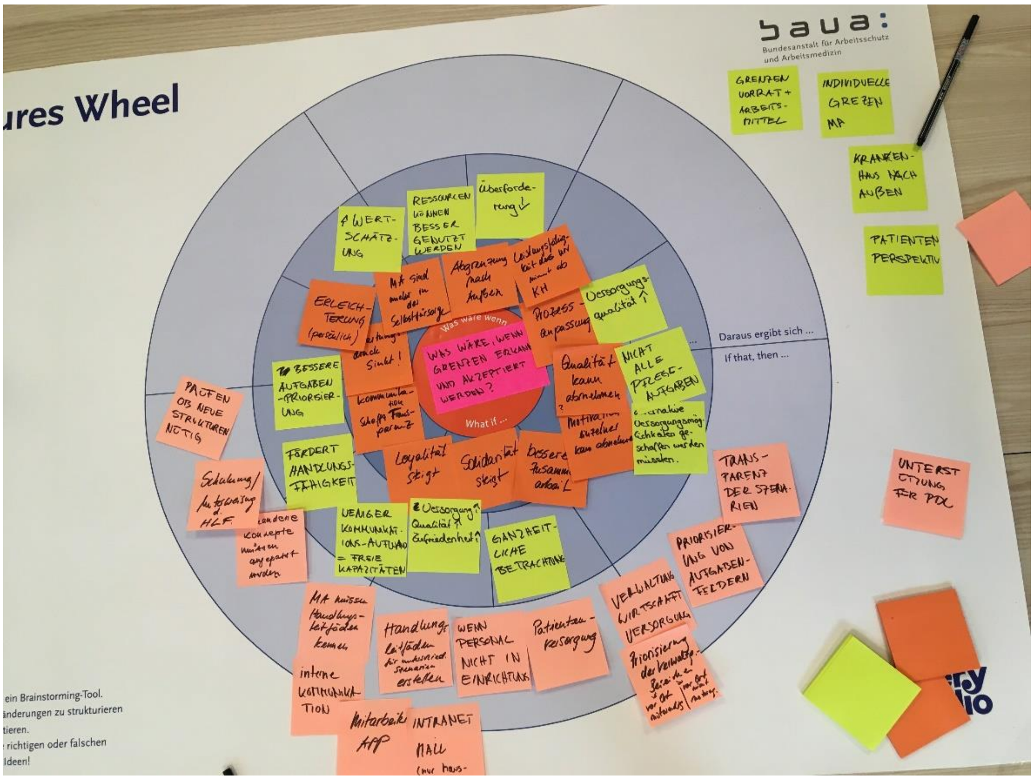

Examples of Ideation Canvases

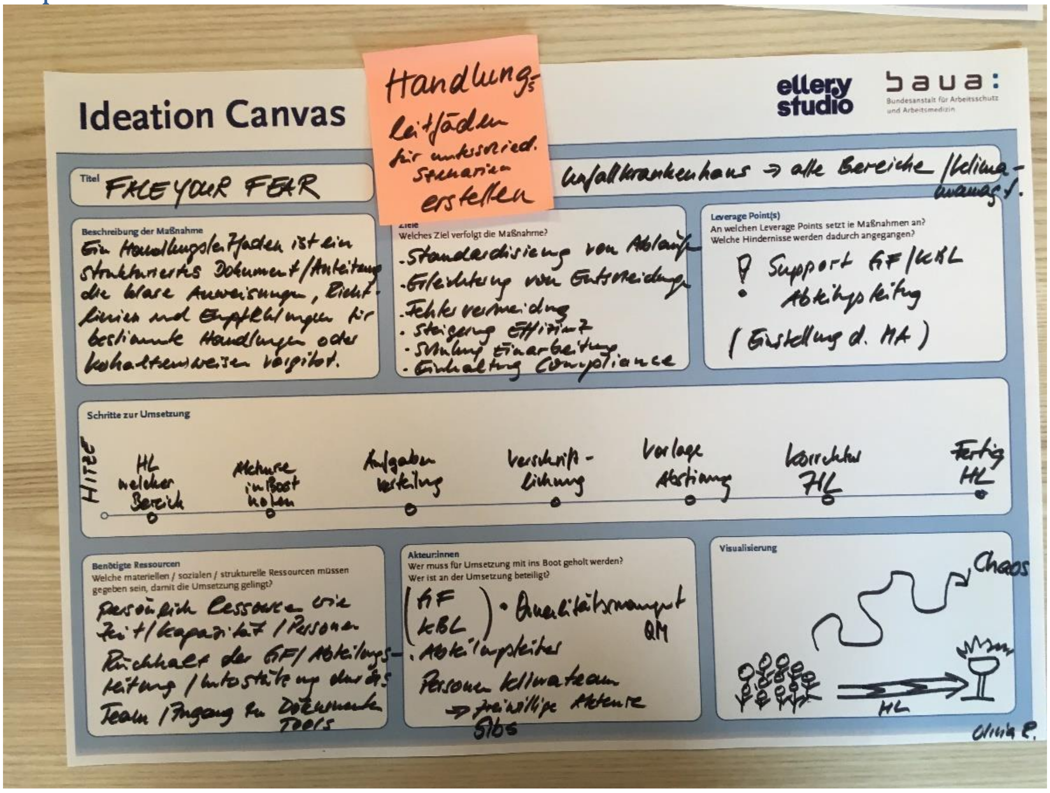

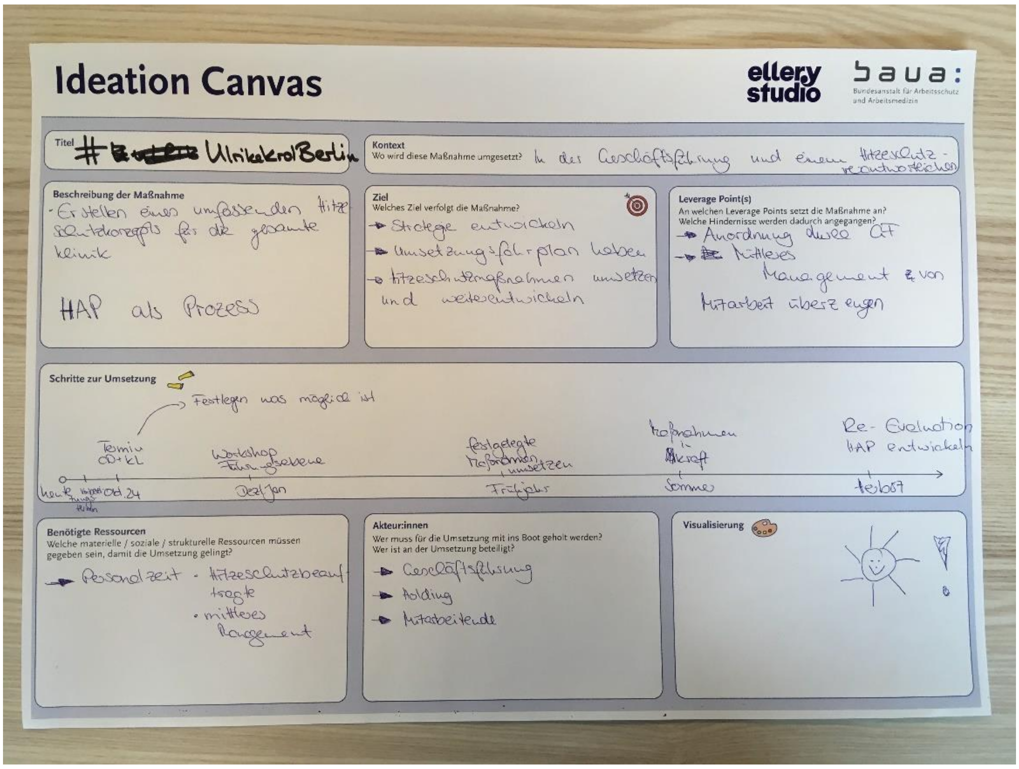

Supplement: Supplementary file 4 [file mmc4.pdf]
