# Supplementary material for: Heat-related challenges and interventions in hospitals: A future-oriented, qualitative approach to improve nurses' working conditions
Source: J Clim Chang Health. 2026 Apr 10;28:100659. doi: 10.1016/j.joclim.2026.100659 (PMC13091379; doi:10.1016/j.joclim.2026.100659)
Supplement: Supplementary file 5 [file mmc5.pdf]

Supplementary File 5

Intervention categorized according to risk- and crisis management cycle

Inhalt

Prevention 1
Preparation 3
Response 3
Recovery 5

Caption: (T) technical interventions, (O) (work-)organizational interventions, (P) personal interventions

Prevention

Staff

- Risk communication: Improve awareness, motivation and health literacy of staff, e.g., through heat scenarios and impact of heat *(P)*
- Management needs to define limits of provision of care during crisis, avoid overwhelming patient loads, and define expectations of nursing staff *(O)*

Patients and provision of care

- Defining and categorizing treatment priorities during crisis and organize alternative treatment in outpatient facilities *(O)*
- Prioritize of nursing tasks: Identify necessary care tasks *(O)*

Communication

- Develop a heat action plan for communicating heat-related interventions and strategies, including guidelines for every profession and hospital areas, with different warning-levels (e.g., elaboration is task of crisis management team) *(O)*
- Communicate performance expectations and limits of provision of care to nursing staff and external stakeholders (e.g., limits of patient admission) *(O)*

- Organizing communication and flow of information to successfully communication during crisis:
  - Develop a staff app for every-day use:
    - obligatory for all employees
    - free of charge
    - real-time access to crisis information
    - access to heat-related protocols, duty rosters, training material at any time
    - enabling team meetings andpeer-to-peer communication
  - Use top-down communication of the heat action plan to ensure hospital-wide knowledge, e.g., through multipliers distributing information *(O)*
  - Facilitate bottom-up communication about the feasibility of interventions, e.g., through middle management and practical trainees *(O)*
  - External communication with regional stakeholders: informing about hospital's heat strategy *(O)*
  - Develop training programs to communicate and ensure practical use of interventions *(P)*
    - Mandatory
    - self-study version
    - documented in app
    - utilization of scenarios
    - basic and role-specific trainings (e.g., for service staff, physicians, nurses)

Resources and infrastructure

- Establish a crisis management team *(O)*:
  - Responsible for hospital's preparedness and communication before, during and after crisis
- Generate financial resources
- Organizing and ensuring resources supply *(O)*:
  - Close cooperation with hospital's device management and purchasing management and operative level to track real-time consumption
  - Ensure internal purchasing structures (e.g., traffic light system, employees to manage supply)
  - Work and elaborate reliable cooperation with external stakeholders to guarantee basic supply
  - Identify all non-essential power consumers to reduce consumption during of short-ages

Other

- Designate cooling zones *(O)*

Preparation

Staff

Adaptation of staffing and working hours *(O)*

- Increase of staff in duty rosters
- Adjustment of working hours
- Reduction in working hours for heat-vulnerable employees

Patients

- Coordinate patient room occupancy (e.g., cooler rooms for vulnerable patients) *(O)*
- Consider core patient admission time for patient transportation, staff, patient and task management *(O)*

Communication

- Create risk awareness and inform staff about prognosis (e.g., via employee app) *(P)*
- Refresh interprofessional training courses *(P)*

Resources and infrastructure

- Provision of cooling appliances (e.g., fans etc.) *(T)*
- Provision of drinks (with minerals) *(O)*
- Stock up supplies *(O)*

Other

- Transfer patient medication to cooler rooms *(O)*
- Decrease indoor temperature (e.g., night time ventilation, including ventilation of unused rooms, early shading) *(O)*

Response

Staff

- Ensure staff rest *(O)*:
  - Increase break regulations and prepare break rooms (e.g., cooling devices, drinks)
- Reduce stress:
  - Restructure work tasks *(O)*
  - Adapt clothing (e.g., light clothing, cold rags), nutrition and fluid intake (e.g., light diet, regular drinking) *(P)*
  - Adjust working hours: share shift work *(O)*
  - Offer individual regulations for heat-vulnerable employees (e.g., sick leave) *(O)*
  - Ensure food supply for employees *(O)*
  - Relocating work, where possible (e.g., home office) *(O)*

Further intensification of situation:

- Provision of psychological employee support (e.g., psychological crisis team) *(O)*
- Organization of accommodation options near the hospital *(O)*

Patients

- Follow care task priority concept *(O)*

- Customize meals (e.g., cooler dishes) *(O)*
- Ensure all necessary and relevant care interventions for patient care *(O)*
- Transfer patients to alternative health providers if overcrowded or patients are experiencing heat stress *(O)*

Communication

- Use internal communication tools for updates on crisis situation, e.g., via employee app *(O)*
- Particularly important: ensure communication with the hospital administration (e.g., via app, emergency staffing) *(O)*

Further intensification of situation:
- Maintain communication to the population: e.g., stay at home *(O)*
- Maintain communication of the crisis team at any time *(O)*

Resources and infrastructure

Ensure hospital access for employees
- Provide rental bikes *(O)*
- Cooperation with public transport *(O)*
- Particularly important: ensure accessibility of the hospital administration *(O)*

Increase staff capacity*(O)*:
- Increase staff with local temporary employment

Further intensification of situation:
- Halt elective surgery program (e.g., only emergency surgeries) *(O)*
- Adapt occupancy management (e.g., closure of rooms or units) *(O)*
- Expand discharge management *(O)*
- Saving and rationing of material*(O)*
  - Particularly important: Save water (e.g., no showering, limit water consumption for employees)
- Provision of further financial resources
- Mutual internal use of materials *(O)*
- Switch off all non-essential power consumers *(O)*
- Expand mobility concept (e.g., scooters, rental cars) *(O)*
- Mutual support with personnel *(O)*
- Cancel or switch off all interventions for patient care that are not necessary (technical and organizational) *(O)*

Other

- Utilize cooling zones *(P)*

Further intensification of situation:
- Identify alternative storage for the deceased individuals if necessary: identify other rooms that can be used (e.g., for laying out or refrigeration) *(O)*

Recovery

Staff

- Focus on staff recovery post-crisis

Other

- Evaluate the heat adaptation plan to improve future responses *(O)*
